# Supplementary material for: Bioactive Metabolites of Marine Origin Have Unusual Effects on Model Membrane Systems
Source: Mar Drugs. 2020 Feb 19;18(2):125. doi: 10.3390/md18020125 (PMC7073740; doi:10.3390/md18020125)
Supplement: Supplementary file 1 [file marinedrugs-18-00125-s001.pdf]

Supplementary Information

For: Jakubec *et al.*, Small additions of bioactive marine sponge metabolites have unusual effects on lipid headgroup mobility and phase organization in membrane model systems

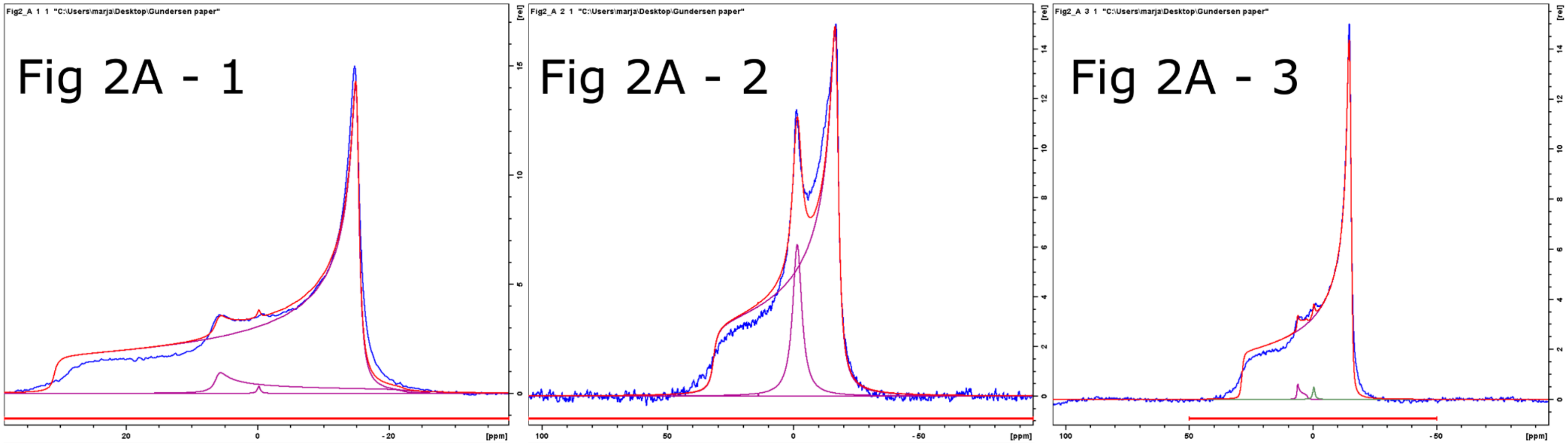

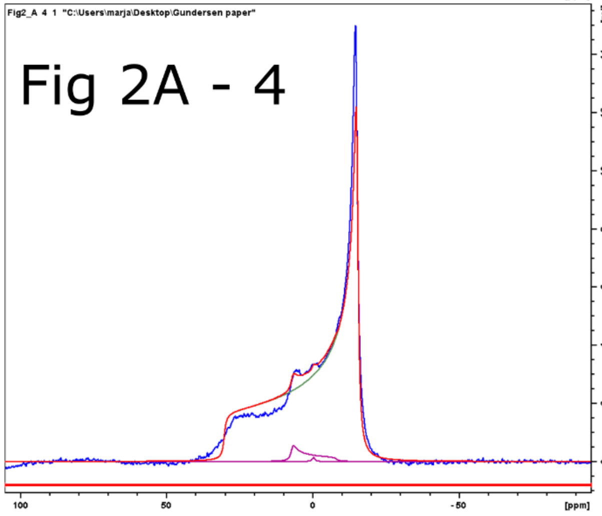

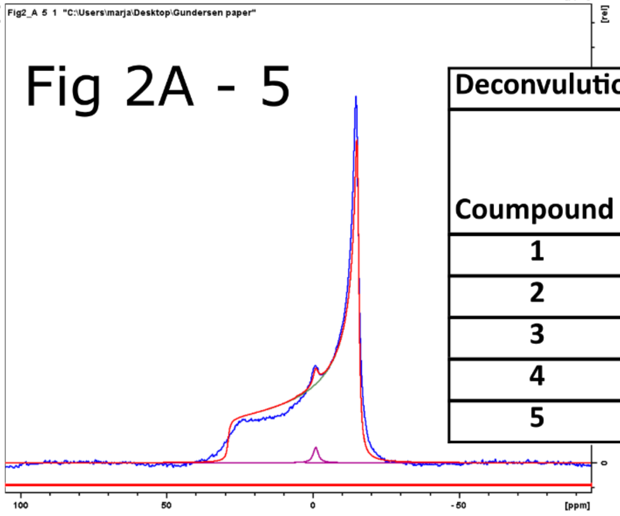

Deconvulution for Fig 2A				
**Coumpound**	**Fluid lamellar phase [%]**	**Inverse hexagonal phase [%]**	**Isotropic phase [%]**	**Overlap of fit [%]**
**1**	93.5	6.4	0.2	93.1
**2**	87.3	0	12.6	92.9
**3**	98.2	1	0.8	95.0
**4**	96.9	3	0.2	91.9
**5**	98.6	0.1	1.3	91.4

Fig. S1. Deconvoluted wide line ^{31}P NMR scans for Fig 3A.

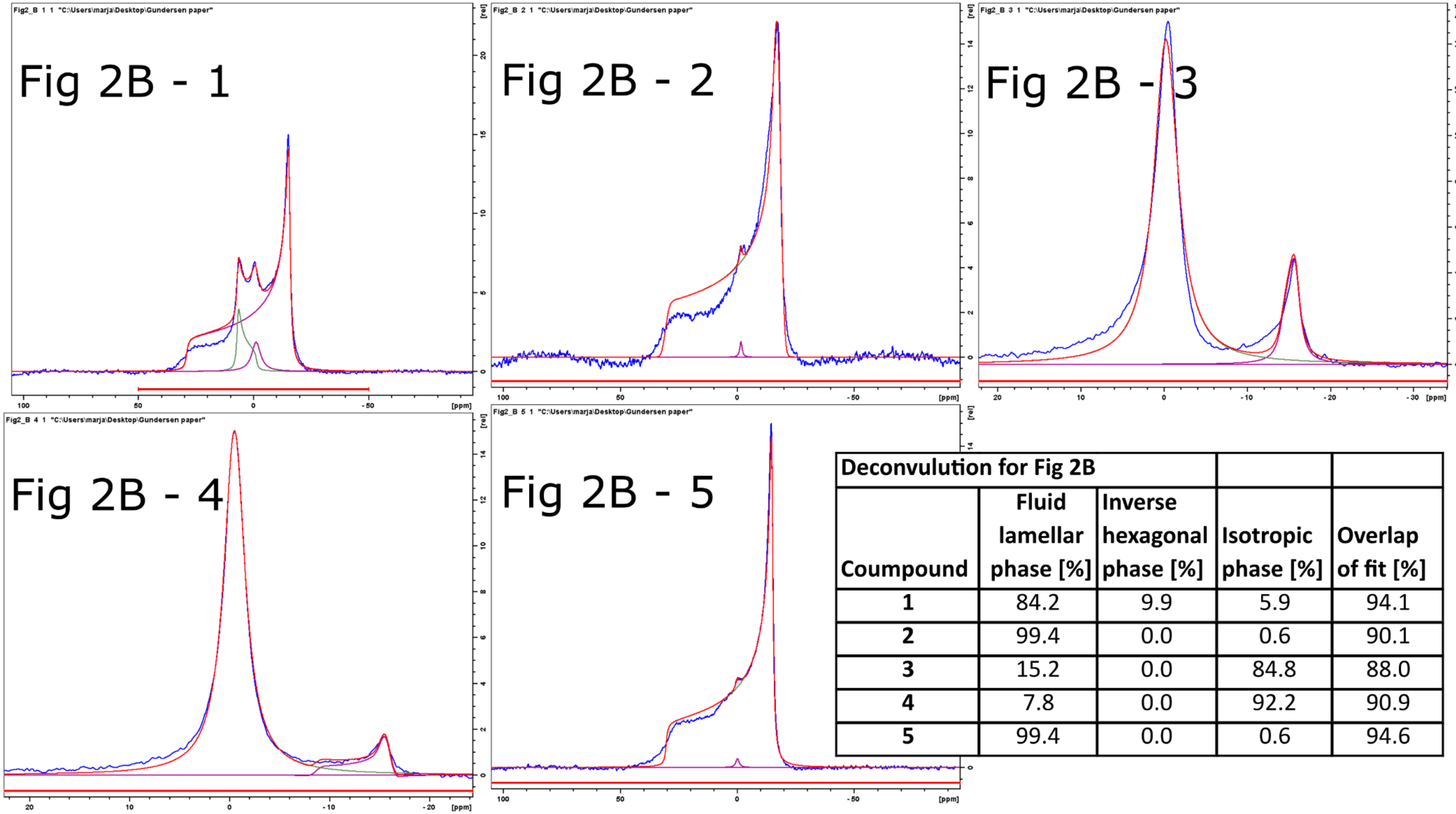

Deconvulution for Fig 2B				
Coumpound	Fluid lamellar phase [%]	Inverse hexagonal phase [%]	Isotropic phase [%]	Overlap of fit [%]
1	84.2	9.9	5.9	94.1
2	99.4	0.0	0.6	90.1
3	15.2	0.0	84.8	88.0
4	7.8	0.0	92.2	90.9
5	99.4	0.0	0.6	94.6

Fig. S2. Deconvoluted wide line ^{31}P NMR scans for Fig 3B.

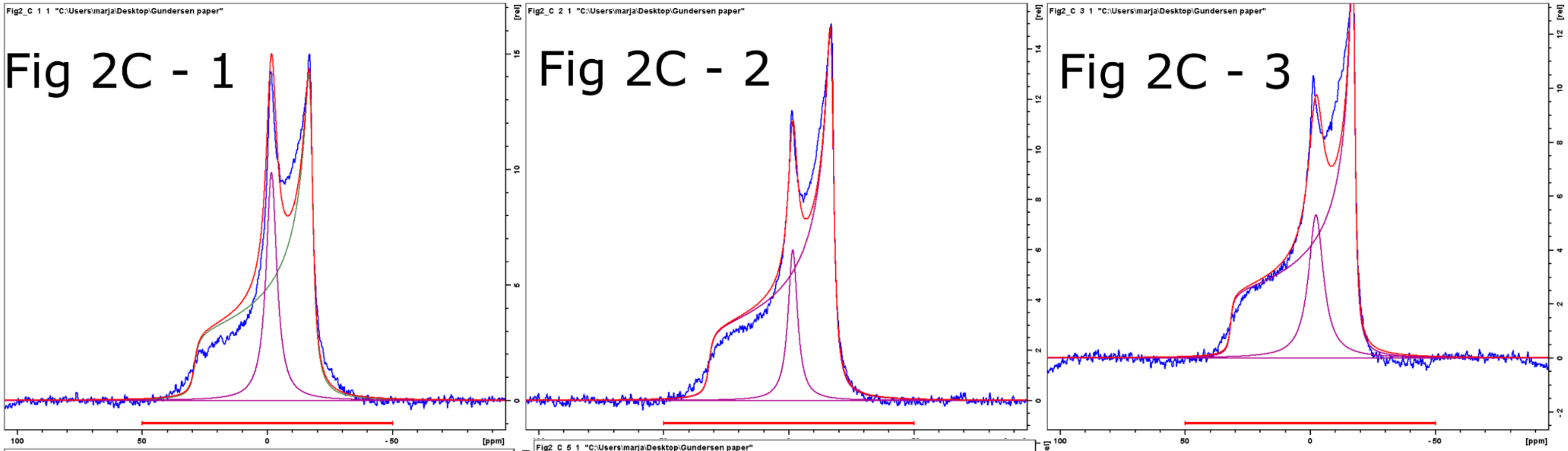

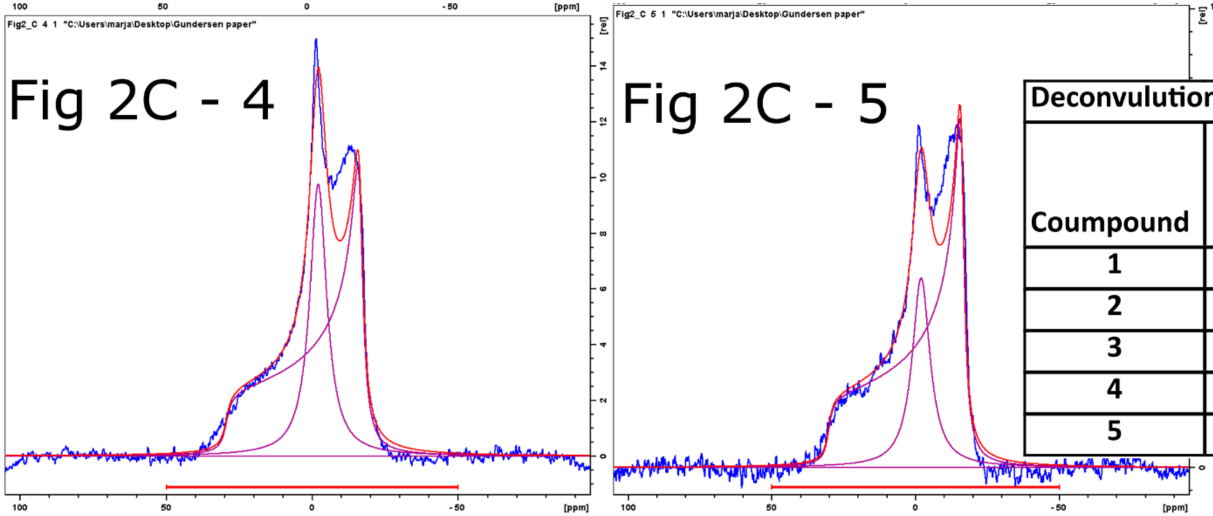

Deconvulution for Fig 2C				
**Coumpound**	**Fluid lamellar phase [%]**	**Inverse hexagonal phase [%]**	**Isotropic phase [%]**	**Overlap of fit [%]**
**1**	87.2	0.0	12.8	94.1
**2**	78.2	0.0	21.8	91.3
**3**	47.6	0.0	52.3	93.8
**4**	67.1	0.0	32.9	93.5
**5**	75.5	0.0	24.5	92.8

Fig. S3. Deconvoluted wide line ^{31}P NMR scans for Fig 3C.

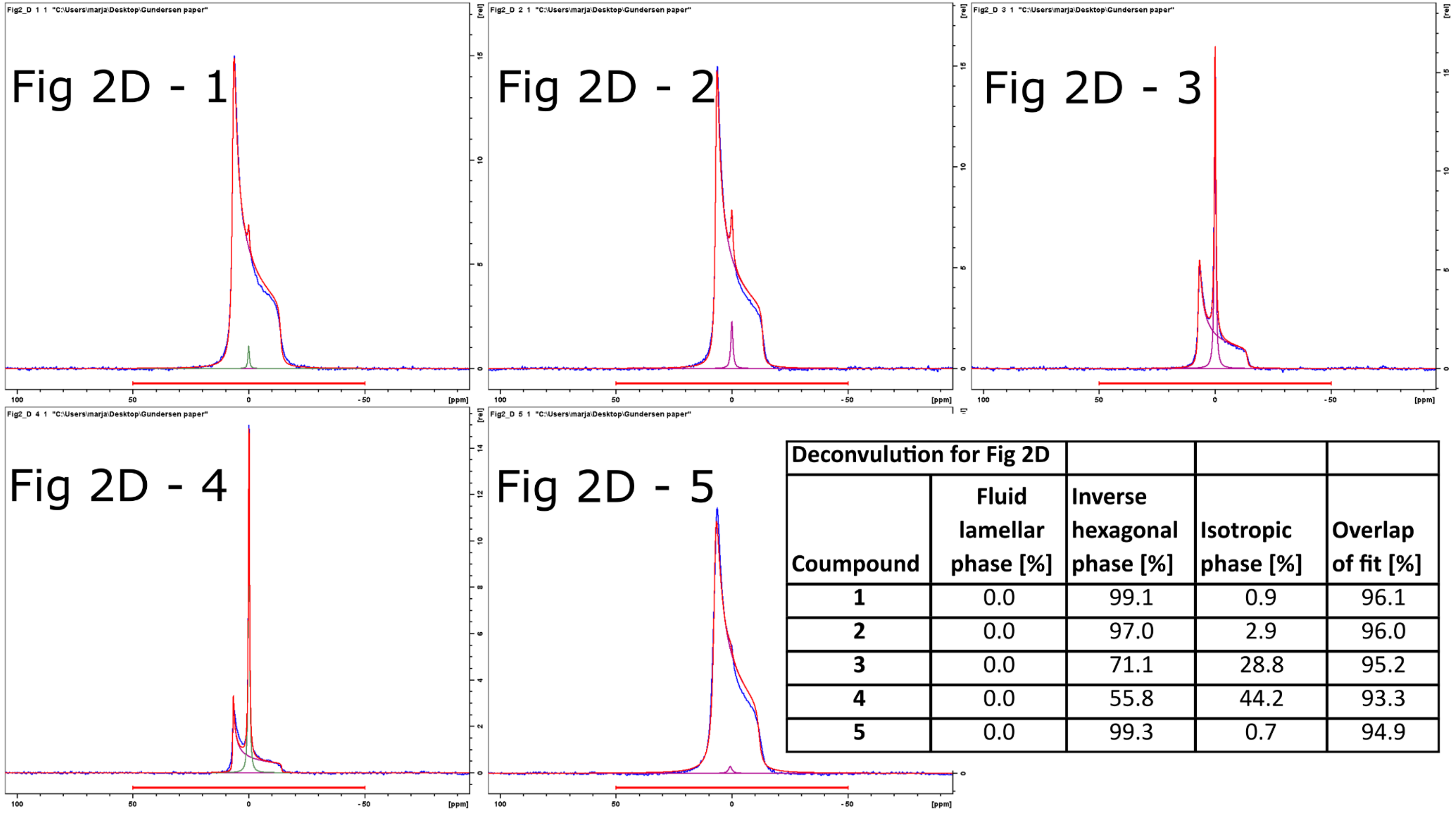

Deconvulution for Fig 2D				
Coumpound	Fluid lamellar phase [%]	Inverse hexagonal phase [%]	Isotropic phase [%]	Overlap of fit [%]
**1**	0.0	99.1	0.9	96.1
**2**	0.0	97.0	2.9	96.0
**3**	0.0	71.1	28.8	95.2
**4**	0.0	55.8	44.2	93.3
**5**	0.0	99.3	0.7	94.9

Fig. S4. Deconvoluted wide line ^{31}P NMR scans for Fig 3D.

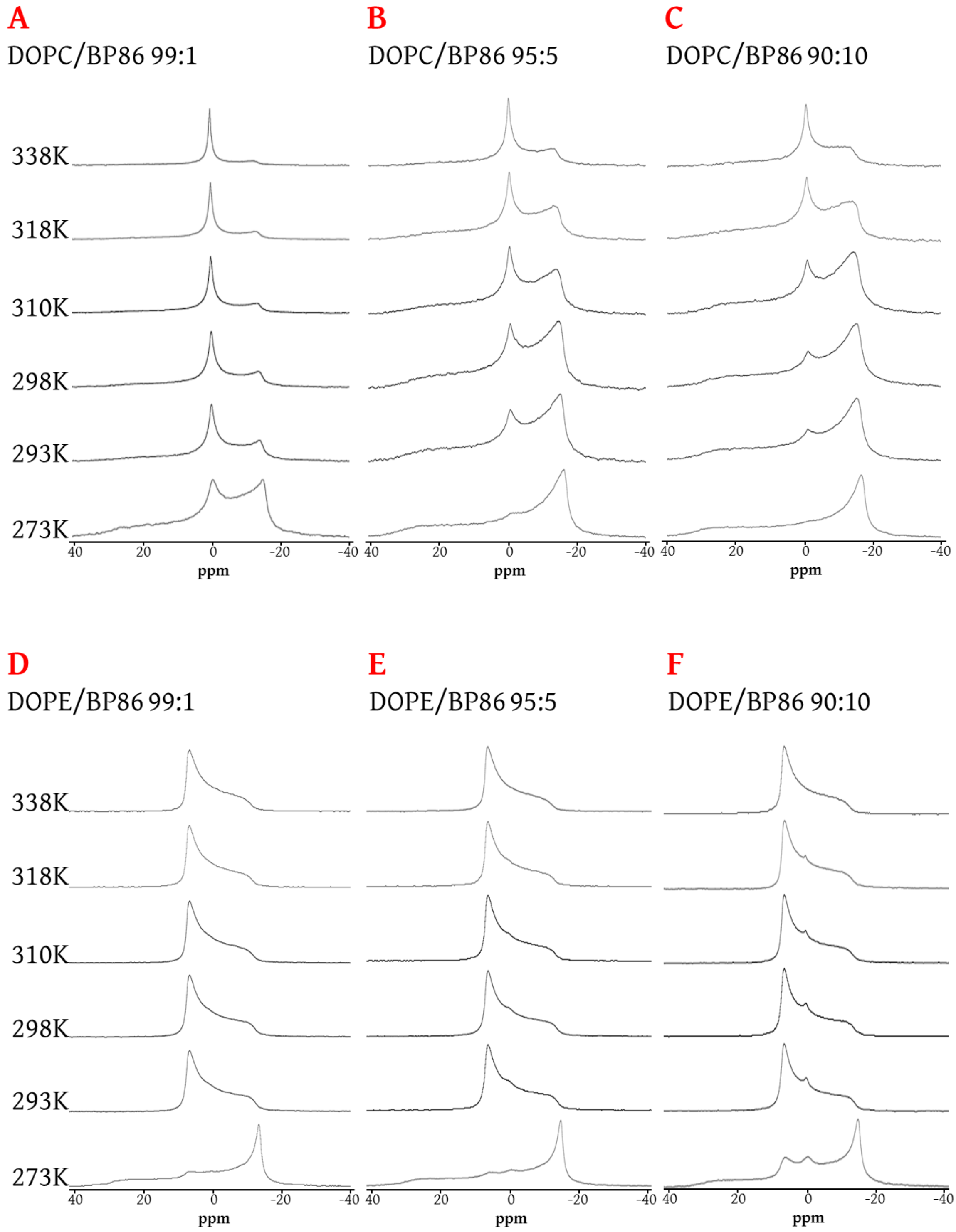

Fig. S5. Wide line ^{31}P NMR temperature scans of DOPC (Panels A-C) and DOPE (panels D-F) doped with compound **1** (BP86; 1-10%).

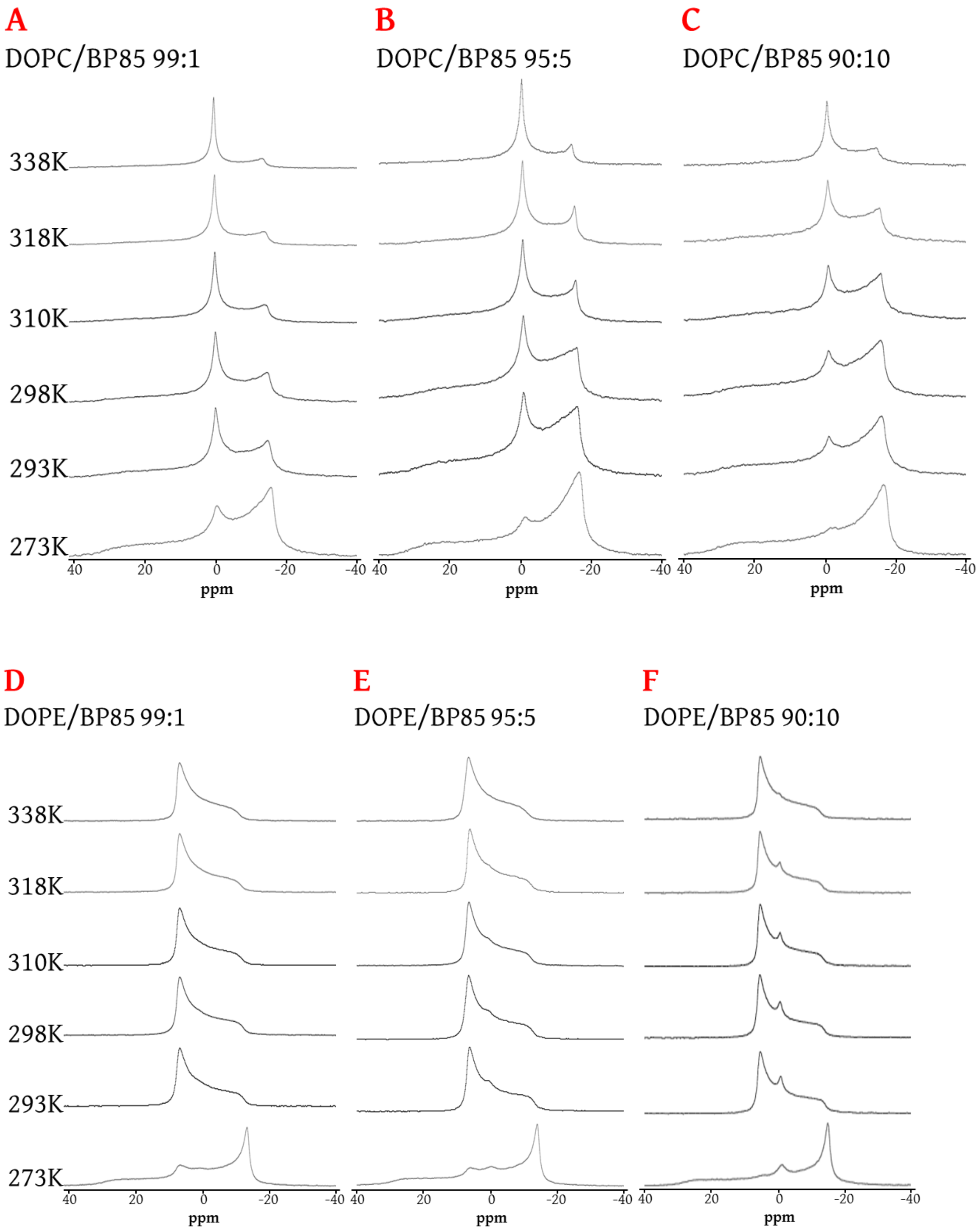

Fig. S6. Wide line ^{31}P NMR temperature scans of DOPC (Panels A-C) and DOPE (panels D-F) doped with compound **2** (BP085; 1-10%).

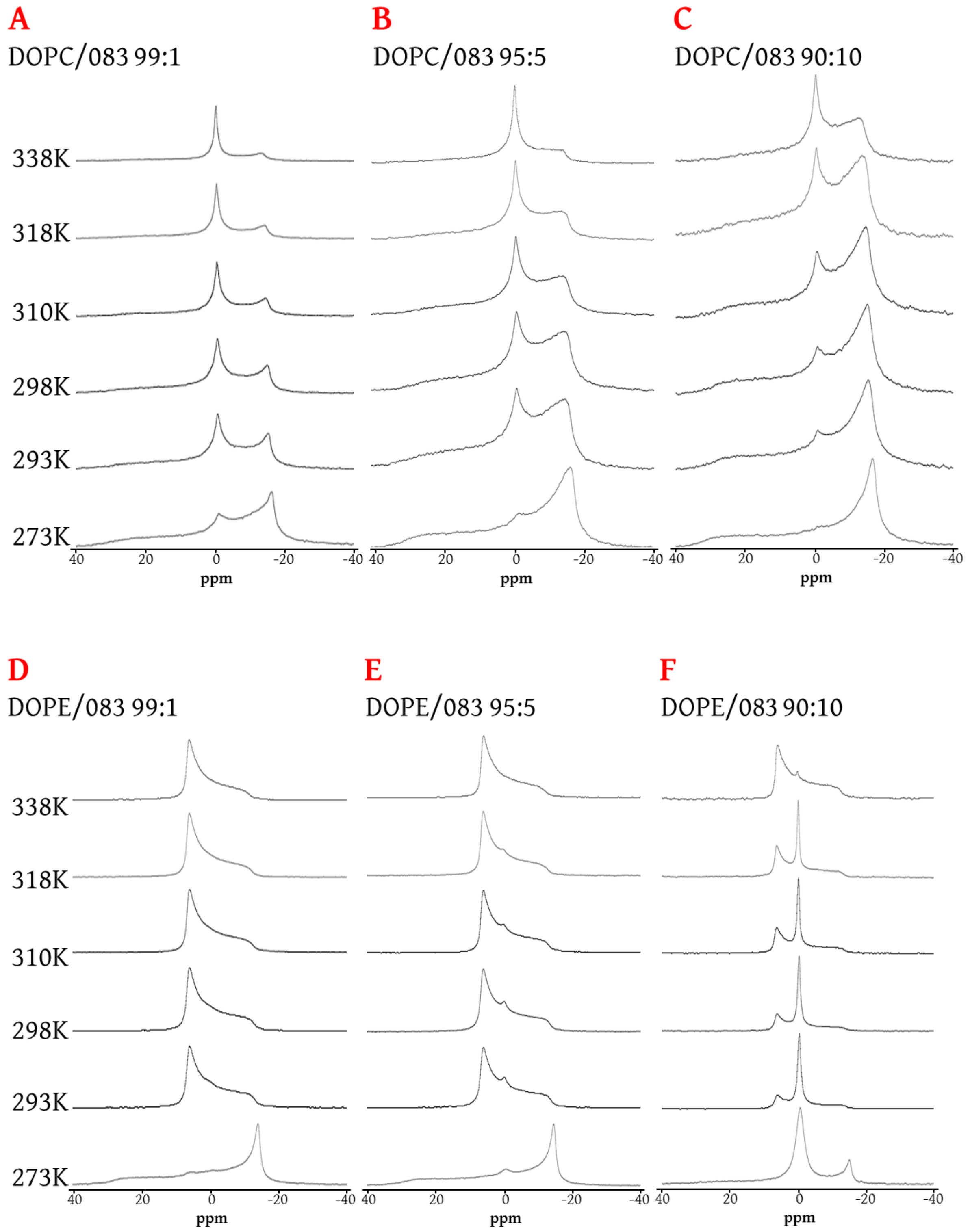

Fig. S7. Wide line ^{31}P NMR temperature scans of DOPC (Panels A-C) and DOPE (panels D-F) doped with compound **3** (083; 1-10%).

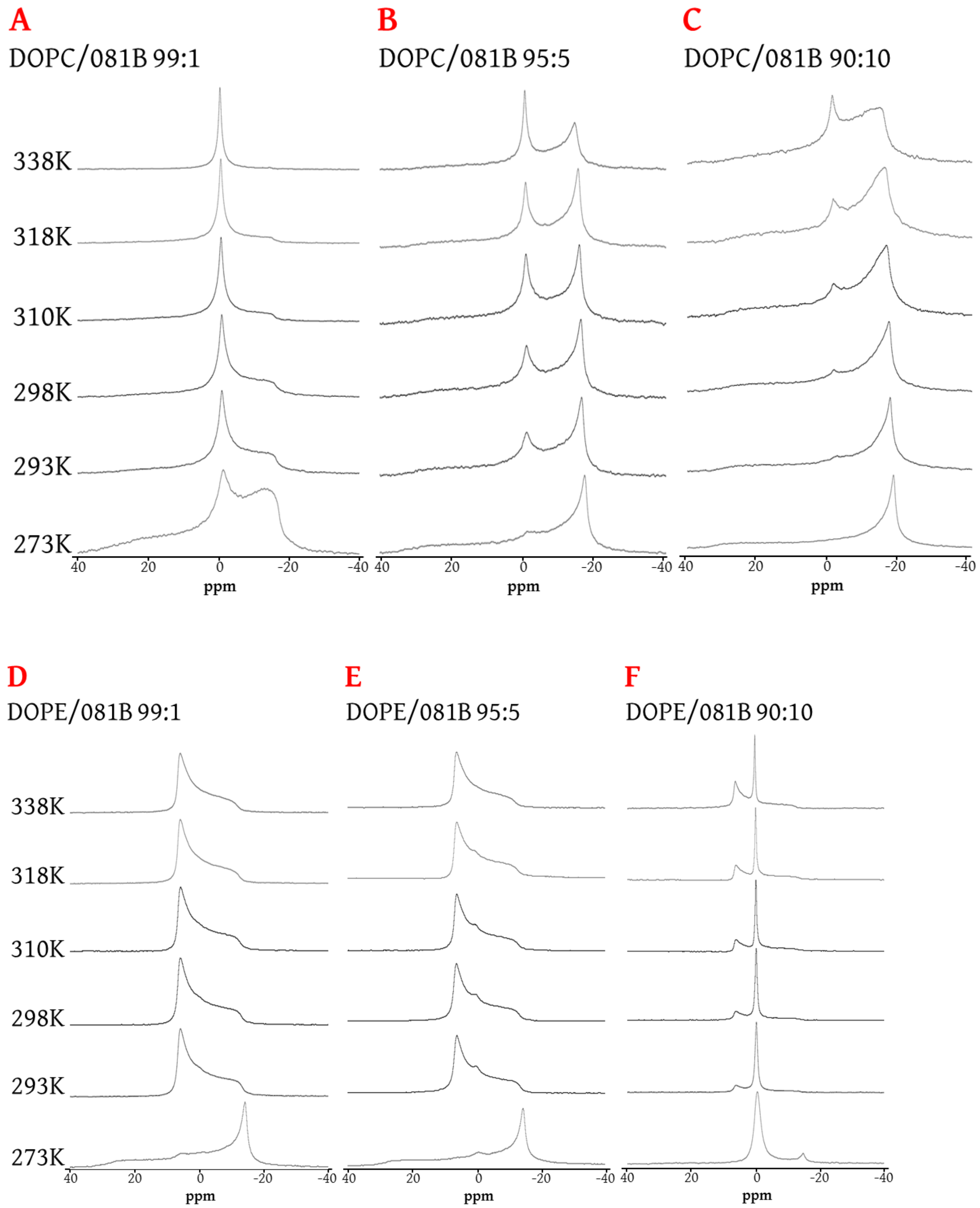

Fig. S8. Wide line ^{31}P NMR temperature scans of DOPC (Panels A-C) and DOPE (panels D-F) doped with compound **4** (081B; 1-10%).

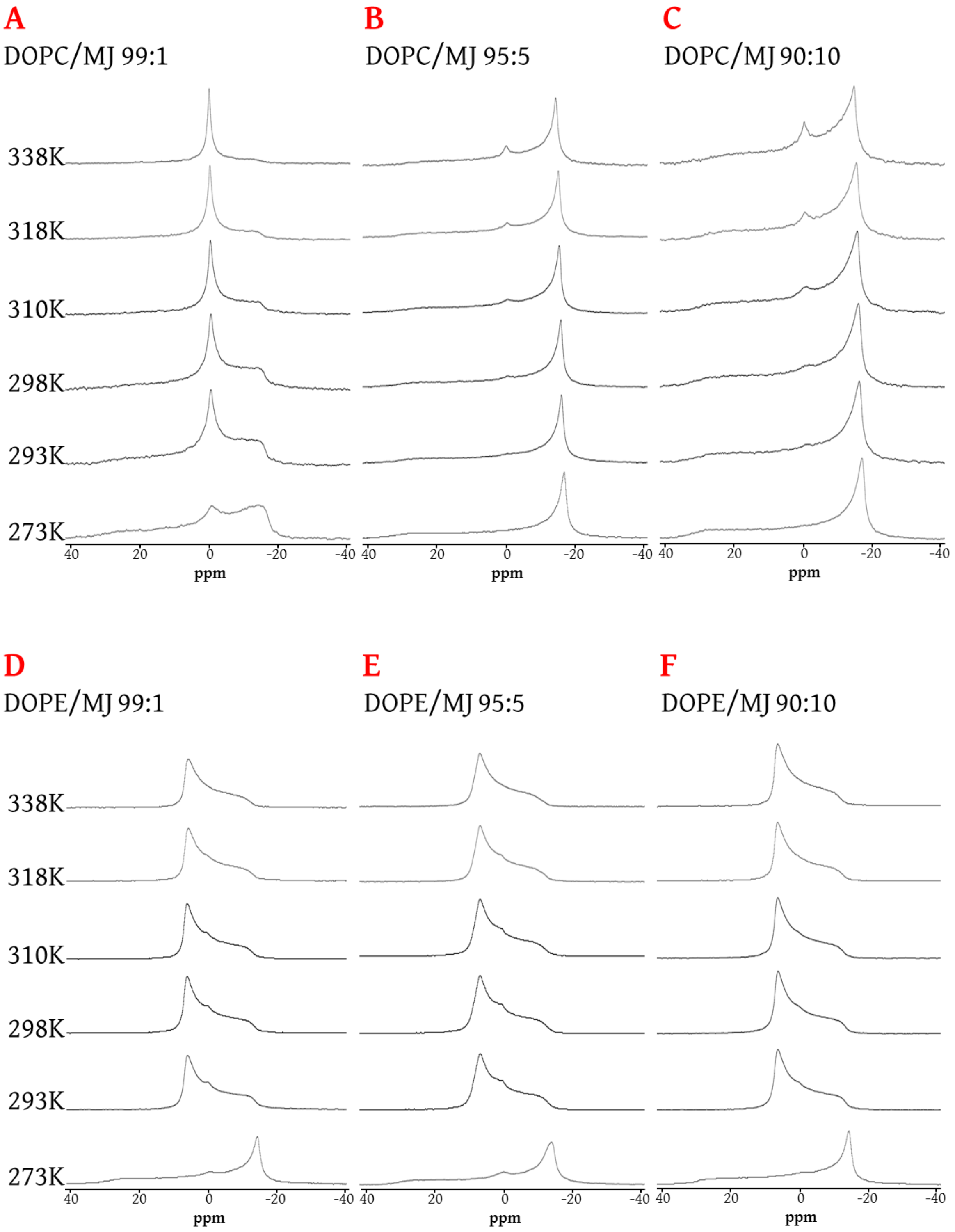

Fig. S9. Wide line ^{31}P NMR temperature scans of DOPC (Panels A-C) and DOPE (panels D-F) doped with compound **5** (MJ; 1-10%).
